# Prospective investigation of autism and genotype-phenotype correlations in 22q13 deletion syndrome and *SHANK3* deficiency

**DOI:** 10.1186/2040-2392-4-18

**Published:** 2013-06-11

**Authors:** Latha Soorya, Alexander Kolevzon, Jessica Zweifach, Teresa Lim, Yuriy Dobry, Lily Schwartz, Yitzchak Frank, A Ting Wang, Guiqing Cai, Elena Parkhomenko, Danielle Halpern, David Grodberg, Benjamin Angarita, Judith P Willner, Amy Yang, Roberto Canitano, William Chaplin, Catalina Betancur, Joseph D Buxbaum

**Affiliations:** 1 Seaver Autism Center for Research and Treatment, Icahn School of Medicine at Mount Sinai, New York, NY, USA; 2 Department of Psychiatry, Icahn School of Medicine at Mount Sinai, New York, NY, USA; 3 Department of Pediatrics, Icahn School of Medicine at Mount Sinai, New York, NY, USA; 4 Department of Neurology, Icahn School of Medicine at Mount Sinai, New York, NY, USA; 5 Department of Neuroscience, Icahn School of Medicine at Mount Sinai, New York, NY, USA; 6 Department of Genetics and Genomic Sciences, Icahn School of Medicine at Mount Sinai, New York, NY, USA; 7 Friedman Brain Institute, Icahn School of Medicine at Mount Sinai, New York, NY, USA; 8 Department of Psychology, St John’s University, Jamaica, NY, USA; 9 INSERM U952, Paris, France; 10 CNRS UMR 7224, Paris, France; 11 Université Pierre et Marie Curie, Paris, France; 12 Mindich Child Health and Development Institute, Icahn School of Medicine at Mount Sinai, New York, NY, USA; 13 Present address: Department of Psychiatry, Rush University Medical Center, Chicago, IL, USA; 14 Present address: Division of Child Neuropsychiatry, General University Hospital of Siena, Siena, Italy

**Keywords:** 22q13 deletion syndrome, Autism, Microarrays, Mutation, Phelan-McDermid syndrome, SHANK3

## Abstract

**Background:**

22q13 deletion syndrome, also known as Phelan-McDermid syndrome, is a neurodevelopmental disorder characterized by intellectual disability, hypotonia, delayed or absent speech, and autistic features. *SHANK3* has been identified as the critical gene in the neurological and behavioral aspects of this syndrome. The phenotype of *SHANK3* deficiency has been described primarily from case studies, with limited evaluation of behavioral and cognitive deficits. The present study used a prospective design and inter-disciplinary clinical evaluations to assess patients with *SHANK3* deficiency, with the goal of providing a comprehensive picture of the medical and behavioral profile of the syndrome.

**Methods:**

A serially ascertained sample of patients with *SHANK3* deficiency (n = 32) was evaluated by a team of child psychiatrists, neurologists, clinical geneticists, molecular geneticists and psychologists. Patients were evaluated for autism spectrum disorder using the Autism Diagnostic Interview-Revised and the Autism Diagnostic Observation Schedule-G.

**Results:**

Thirty participants with 22q13.3 deletions ranging in size from 101 kb to 8.45 Mb and two participants with *de novo SHANK3* mutations were included. The sample was characterized by high rates of autism spectrum disorder: 27 (84%) met criteria for autism spectrum disorder and 24 (75%) for autistic disorder. Most patients (77%) exhibited severe to profound intellectual disability and only five (19%) used some words spontaneously to communicate. Dysmorphic features, hypotonia, gait disturbance, recurring upper respiratory tract infections, gastroesophageal reflux and seizures were also common. Analysis of genotype-phenotype correlations indicated that larger deletions were associated with increased levels of dysmorphic features, medical comorbidities and social communication impairments related to autism. Analyses of individuals with small deletions or point mutations identified features related to *SHANK3* haploinsufficiency, including ASD, seizures and abnormal EEG, hypotonia, sleep disturbances, abnormal brain MRI, gastroesophageal reflux, and certain dysmorphic features.

**Conclusions:**

This study supports findings from previous research on the severity of intellectual, motor, and speech impairments seen in *SHANK3* deficiency, and highlights the prominence of autism spectrum disorder in the syndrome. Limitations of existing evaluation tools are discussed, along with the need for natural history studies to inform clinical monitoring and treatment development in *SHANK3* deficiency.

## Background

22q13 deletion syndrome, also known as Phelan-McDermid syndrome, is a genetic disorder characterized by global developmental delay, hypotonia, delayed or absent speech, and autistic behaviors [1]. *SHANK3* is the critical gene for the core neurological and behavioral symptoms in this syndrome, as the loss of one copy (haploinsufficiency) of *SHANK3*, occurring through intragenic deletion or point mutation, is sufficient to cause the neurobehavioral manifestations of Phelan-McDermid syndrome [2-5]. *SHANK3* codes for a master scaffolding protein that forms a key framework in the postsynaptic density of glutamatergic synapses and plays a critical role in synaptic function, learning and memory [6].

*SHANK3* deletion or mutation (which we will refer to together as *SHANK3* deficiency) is found in about 0.5% of patients ascertained for autism spectrum disorder (ASD), including 0.2% with a *SHANK3* mutation identified by sequencing [2-4,7,8] and 0.3% with a *SHANK3* deletion, as shown by microarray analyses of over 7,000 individuals with ASD([3,9-15], Autism Genome Project, unpublished). Analysis of a very large cohort of patients with intellectual disability (ID) also indicates that about 0.3% of such patients have *SHANK3* deletions [16], with some studies reporting rates above 1% [17]. The rate of *SHANK3* mutation in ID is still being determined, but the first two studies estimate it at approximately 1% [17,18]. These findings suggest that *SHANK3* deficiency is one of the more common monogenic causes of ASD and ID. Furthermore, recent evidence suggests that disruption of the *SHANK3* and glutamate signaling pathway is common to multiple forms of ASD, including Fragile X syndrome and tuberous sclerosis [19,20]; dissecting this pathway may therefore represent an important opportunity to improve understanding of the biological pathways associated with ASD and ID not involving haploinsufficiency of *SHANK3*.

More than 150 affected individuals with *SHANK3* deficiency have been described in published case studies since the first case report by Nesslinger and colleagues in 1994 [21]. These cases suggest a common underlying phenotype that includes global developmental delay, severe expressive language delay, hypotonia, autistic features and minor dysmorphic features [21-30]. However, clinical and genetic methodology varied across studies, as did estimates of the nature and prevalence of ASD, relying in the majority of cases on parental reports or questionnaires. These reports highlight a broad and clinically heterogeneous phenotype. Dysmorphic features are commonly described and include dysplastic toenails; dysplastic ears; large, fleshy hands; long eyelashes; dolichocephaly; pointed chin; and bulbous nose [21-26,28,29]. Medical conditions associated with the syndrome are less well defined but have been reported to include seizures, renal abnormalities, cardiac defects, hearing loss, gastroesophageal reflux and lymphedema [23-25,28].

The first explicit association between ASD and *SHANK3* deficiency was published in 2000 by Prasad and colleagues, who described three cases of individuals with pervasive developmental disorder and terminal 22q13 deletion [31]. Among the case series published since then, several have specifically evaluated the presence of ASD using a variety of measures such as medical record review [24,26], developmental questionnaires [23] and various standardized diagnostic instruments [22,32]. Likely because of the diverse approaches, estimates of rates of ASD vary dramatically across studies. A review of 107 cases previously described in the literature reported a rate of ‘autistic behaviors’ of 44% [33]. Studies that prospectively evaluate ASD in patients with 22q13 deletions and utilized standardized assessments (for example, Childhood Autism Rating Scale, Social Communication Questionnaire) suggest rates of 60% to 94% [22,25,28]. Philippe *et al*. [27] administered the Autism Diagnostic Interview-Revised (ADI-R, [34]) in eight participants and found that, although none met criteria for an autism classification, five (63%) met cutoff scores in the social and communication domains and were within one point of cutoff scores on the repetitive behavior domain, suggesting a level of autistic features consistent with Diagnostic and Statistical Manual of Mental Disorders - Fourth Edition (DSM-IV) diagnoses of pervasive developmental disorder. To date, published investigations have not evaluated ASD in *SHANK3* deficiency using best practice recommendations, which include combining information from clinician evaluations, structured observation, and an autism-focused, structured developmental history [35]. As such, the nature and prevalence of ASD among patients with *SHANK3* deficiency remains an important area of study.

Evaluations of medical comorbidities associated with *SHANK3* deficiency have been likewise limited to primarily retrospective reports. Electroencephalography (EEG) recordings have never been prospectively collected in published studies of 22q13 deletion syndrome, although five case series report on the prevalence of seizure disorders using retrospective review and parent survey methods [22,23,25,27,28]. These prior prevalence estimates of seizure disorders range from 0% (out of 8) [27] to 31% (4 out of 13) [28], depending on the study.

Among the published case series, 31 patients have had brain magnetic resonance imaging (MRI) results described, either through prospective assessment [27,28,36] or medical record review [24]. Brain abnormalities were evident in 22 out of 31 (71%) cases, ranging from 2 out of 4 (50%) [24] to 10 out of 10 (100%) [36]. Changes in the corpus callosum were described in 16 out of 31 patients, including thinning or hypoplasia; 14 had white matter changes, including delayed myelination, generalized white matter atrophy and nonspecific white matter hyperintensities; 12 had ventricular dilatation; and 3 had interventricular, cerebellar or temporal sylvian arachnoid cysts. The first case series in *SHANK3* deficiency reported that four out of seven patients had ventricular dilatation [21] although methods were not specified. The most recent study specifically examined cerebellar malformations in 10 patients with 22q13 deletions using MRI and found evidence of cerebellar vermis hypoplasia in six patients, including an enlarged cisterna magna in five [36].

The nature and prevalence of other clinical features (for example, cognitive impairment, language impairment and motor deficits) have also not been systematically evaluated in some of the previous studies. Phillipe *et al*. [27] conducted prospective neuropsychological evaluations of eight children with 22q13 deletions and present a rich clinical picture of neurocognitive features, particularly in the motor domain. The authors reported mild delays in acquisition of early motor milestones and two patterns of motor disabilities: orthopedic disorders (including hypotonia of lower limbs) and impairments in motor control (for example, praxis, pyramidal signs and postural abnormalities).

The present study seeks to expand upon the existing literature by contributing results from systematic evaluations of phenotypic domains in patients with *SHANK3* deficiency using a prospective design and standardized evaluation methods. Detailed clinical and molecular analyses represent an important opportunity to improve the understanding of underlying biological pathways associated with ASD, and neurodevelopmental disorders more broadly. In addition, the knowledge gained from comprehensive evaluations may help to establish a neurobiological signature of the disorder and serve to identify important phenotypic targets for treatment intervention or candidate biomarkers of treatment response. Finally, results from *SHANK3* sequencing and high-resolution chromosomal microarray analysis (CMA) may also provide insight into the relationship between genotype and phenotype in this complex syndrome.

## Methods

### Participants

Participants were enrolled into an institutional review board-approved project with parents providing informed consent for participation. Over an 18-month period, 32 children and adults (18 males and 14 females) were seen. Referral sources included the Phelan-McDermid Syndrome Foundation, on-going research studies and word-of mouth. Patients ranged in age from 1.6 to 45.4 years (mean 8.8, SD 9.2). All patients were of Caucasian descent. Phenotypic data were available on all participants with rates of 90% to 100% completion on diagnostic, cognitive and clinical genetics evaluations. Half of the participants received structured neurological examinations by a neurologist, but all patients were assessed for neurologic abnormalities during the clinical genetics evaluation.

### Clinical evaluation

An inter-disciplinary team evaluated patients using the following clinical tools:

1. *Psychiatric evaluations* using the DSM-IV [37] were conducted by board-certified child and adolescent psychiatrists at the first patient visit and focused on the assessment of pervasive developmental disorders.

2. *Clinical genetics evaluations* and dysmorphology examinations were performed by clinical geneticists to assess growth, pubertal development, head size, craniofacial features, digits, extremities, chest, spine, skin, organ malformations (such as congenital heart or renal defects) and neurological abnormalities.

3. *Neurological examinations* were conducted by a pediatric neurologist to evaluate gross motor skills and gait, fine motor coordination, cranial nerves and deep tendon reflexes.

4. *Autism Diagnostic Observation Schedule-G* (ADOS-G [38]), a direct semi-structured assessment, was used to assess the presence of the three main domains of autism (communication, reciprocal social interaction and repetitive, restricted behaviors). ADOS-G, Module 1, intended for minimally verbal individuals, was administered by a trained clinician to all patients in this sample.

5. *ADI-R*[34], an investigator-based, semi-structured instrument, was used to differentiate autistic disorder from non-autistic ID in individuals with a developmental age greater than 18 months. It was administered by a trained interviewer to parents or caregivers, making use of an algorithm that incorporates the World Health Organization International Classification of Diseases-10 and DSM-IV criteria for diagnosis.

6. *Cognitive testing* was conducted by licensed clinical psychologists or doctoral students to provide estimates of cognitive functioning. Tests were selected based on age of child and developmental level. The Mullen Scales of Early Learning [39] were used on 27 participants, including children with chronological ages older than the standardization sample, to allow for flexible administration of items. The Stanford-Binet Intelligence Scales, Fifth Edition [40] were used for one school-aged child who used single words on a regular basis, and the Leiter International Performance Scale-Revised [41] was used on two adult patients. Ratio intelligence quotients (IQs) were calculated using mental age estimates from the cognitive tests and used to provide an estimate of nonverbal IQ.

7. The *Vineland Adaptive Behavior Scales, Second Edition*[42] were used to evaluate independence in daily life skills, including communication, socialization and motor skills.

8. *Medical record reviews* were conducted to ascertain information on medical, neurological and physical manifestations not directly assessed in the clinical evaluations listed above. Results from EEG and brain MRI were collected from record review when available.

### Genetic testing

CMA and multiplex ligation-dependent probe amplification (MLPA) were used to confirm previous clinical genetic testing in 29 out of 30 patients with 22q13 deletions. DNA from one participant (SH27) was not available for in-house validation, so the clinical laboratory CMA report using the Affymetrix 6.0 platform was reviewed to confirm loss of *SHANK3*. Two patients (SH29 and SH32) had deleterious *de novo* point mutations in *SHANK3*, and Sanger sequencing was carried out to confirm the mutation (see below).

MLPA probe set SALSA P188 MLPA kit 22q13 from MRC-Holland (Amsterdam, the Netherlands) was used according to the manufacturer’s protocols. A total of 37 probes are on 22q13 and four of them on the *SHANK3* gene. Briefly, 200 ng genomic DNA in 5 μl water was denatured, mixed with the probe set and high salt MLPA buffer. The mixture was first hybridized at 60°C for 16 h, followed by a ligation reaction; 5 μl of the ligation reaction product was used for PCR. PCR products were subjected to capillary electrophoresis on an ABI 3130 genetic analyzer (Applied Biosystems, Foster City, CA, USA). Raw traces from the electrophoresis were imported into GeneMarker software (SoftGenetics, State College, PA, USA) for MLPA analysis. After population normalization, data were compared with a synthetic control sample, which represents the median of all normal samples in the experiment. A threshold of dosage change <0.75 was used to identify deletions, and a threshold >1.30 was used to identify duplications.

Twenty-nine samples were genotyped on Illumina Omni 2.5-8 v1 array (Illumina, San Diego, CA, USA) containing a total of 2,379,855 markers. Samples were processed according to the manufacturer’s protocol and normalization and genotype calling were performed using Illumina Genome Studio software V2011.1. All samples had an array call rate above 99%, SD for log R ratio values in the autosomes <0.28 and SD for B allele frequency values <0.06. Copy number variants (CNVs) were called using PennCNV algorithm [43]. Fragmented CNVs were merged and the breakpoints were inspected by visual investigation of log R ratio and B allele frequency values within and in the vicinity of the called CNVs. The exact deletion breakpoints in the 22q13 region were confirmed using the moving average of the number of markers with B allele frequency between 0.45 and 0.55, in addition to the visual investigation of the PennCNV call. Genomic locations are based on GRCh37 (hg 19).

*SHANK3* point mutations were identified in two patients, SH32 had a nonsense mutation (c.1527G>A, p.W509*) in exon 12 and SH29 had a frameshift single base deletion (c.2497delG, p.P834Rfs*59) in exon 21 of the *SHANK3* gene (NM_033517.1). As we reported previously [44], because the current version of reference human genome assembly GRCh37 misses the beginning of exon 11, the cDNA and amino acid positions here have been corrected according to the most updated *SHANK3* mRNA and protein sequence (NM_033517.1 and NP_277052.1) in RefSeq. PCRs were designed to amplify the corresponding target fragments from 20 ng genomic DNA of each family member using AccuPrime Pfx (Invitrogen, Carlsbad, CA, USA) in a 10 μl total PCR reaction volume. Two DNA fragments were amplified: a 389-bp fragment covering exon 12 (forward primer 5′-CGAGCCCATCTGTTCCTTT-3′, reverse primer 5′-TCCATGTGGATTTAGCACCA-3′), and a 264-bp exonic fragment covering exon 21 (forward primer 5′-GAGCACCTCGATGCAAGAC-3′, reverse primer 5′-TTCTGCCGCTCGGGATAC-3′). The amplified DNA fragments were then purified and sequenced on an ABI 3730 DNA analyzer using the BigDye Terminator v3.1 Cycle Sequencing kit (Applied Biosystems).

### Data analysis

Descriptive statistics were calculated across all measures and organized into phenotypic domains: medical comorbidities, dysmorphic features, behavioral and ASD features, cognitive ability, receptive and expressive language, and motor skills. For genotype-phenotype analyses, the deletion size for the two patients with point mutations was entered as the size of the *SHANK3* gene, 58,572 bp. Spearman rank order correlations (SROC) were used to explore associations between deletion size and continuous phenotyping variables (for example, number of medical comorbidities, number of dysmorphic features, IQ). Pairwise deletion of missing cases was used in correlation analysis to address missing data in the SROC calculations. *P*-values were calculated by bootstrapping 95% confidence intervals around the bootstrap-estimated SROC, with bias corrected and accelerated confidence interval reported below. Mann–Whitney U tests were conducted to explore potential associations between each medical comorbidity and dysmorphic features (dichotomous variables) with the continuous but skewed measure of deletion size.

## Results

We carried out comprehensive, prospective clinical evaluations of 32 patients with a 22q13 deletion (n = 30), *SHANK3* mutation (n = 1), or ascertained with ASD and subsequently identified by whole exome sequencing as having a *de novo SHANK3* mutation (n = 1).

### Genetic testing

**Figure 1:**
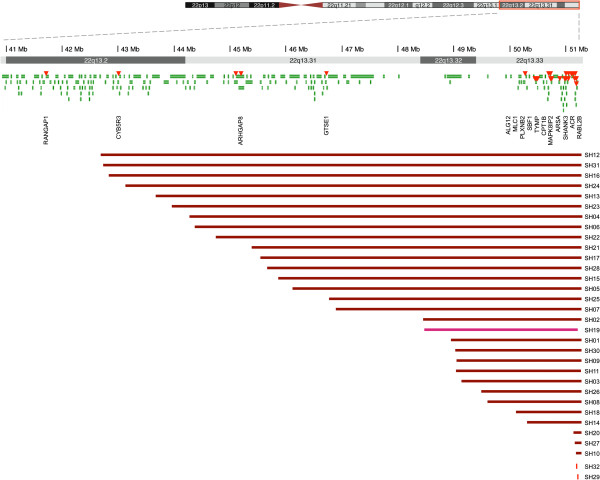
**Deletion sizes at 22q13.2-11q13.3 in 32 patients.** Genes are indicated across the top in green, the red triangles represent MLPA probes. The two patients with *SHANK3* mutations are shown in red and the patient with an interstitial deletion is shown in pink. Genomic coordinates correspond to the hg19 genome assembly (Build 37).

As shown in Table 1, all 32 participants had confirmed *SHANK3* deficiency secondary to mutation or deletion. Two participants had single base-pair changes leading to a premature stop, either due to a nonsense change (c.1527G>A) or a frameshift (c.2497delG). Among the 30 patients with deletions, six had ring chromosome 22, two had unbalanced translocations with loss of distal 22q, and one had an interstitial deletion that included *SHANK3*; the remaining 21 participants (66%) had simple terminal deletions of 22q. Deletion sizes ranged from 101 kb to 8.45 Mb (mean ±SD, 4.21 ±2.75 Mb) (Figure 1). Deletions and mutations were confirmed to be *de novo* in 25 parent dyads. Samples from three fathers and three mothers from an additional six participants were studied and did not carry deletions of *SHANK3*. All parents were healthy, suggesting a *de novo* origin for those remaining. In one patient for whom blood could not be obtained for further molecular characterization (SH27), the affected child had a 22q terminal deletion based on a clinical laboratory report. The patient’s mother has a balanced translocation, 46,XX, t(11;22)(q23;q11.2); the child’s karyotype was normal.

**Table 1:** **Description of the genetic changes in 32 participants with 22q13 deletions or *SHANK3 *mutations**

**Patient**	**Gender**	**Ascertainment method**	**Rearrangement**	**Del 22q13, array coordinates (hg19)**	**Del 22q13 size**	**Validation**	**Inheritance**	**Additional genomic findings**
SH1	Male	Karyotype, FISH	Ring 22	48927548-51224208	2,296,661	MLPA	Mother negative	None
SH2	Male	Karyotype, aCGH	Ring 22	48444959-51224208	2,779,250	MLPA	*De novo*	None
SH3	Male	Karyotype, SNP array	Ring 22	49114430-51224208	2,109,779	MLPA	*De novo*	None
SH4	Female	aCGH	Terminal deletion	44321641-51224208	6,902,568	MLPA	*De novo*	None
SH5	Male	aCGH	Terminal deletion	46143471-51224208	5,080,738	MLPA	*De novo*	None
SH6	Female	aCGH, FISH	Terminal deletion	44427703-51224208	6,796,506	MLPA	*De novo*	None
SH7	Female	Karyotype, FISH	Ring 22	46905533-51224208	4,318,676	MLPA	Mother negative	None
SH8	Female	aCGH, and FISH	Terminal deletion	49574124-51224208	1,650,085	MLPA	*De novo*	None
SH9	Female	aCGH, FISH	Terminal deletion	49028732-51224208	2,195,477	MLPA	*De novo*	None
SH10	Male	SNP microarray	Terminal deletion	51122946-51224208	101,263	MLPA	*De novo*	None
SH11	Female	aCGH, FISH	Terminal deletion	49028732-51224208	2,195,477	MLPA	*De novo*	None
SH12	Male	aCGH	Terminal deletion	42773732-51224208	8,450,477	MLPA	*De novo*	None
SH13	Female	Karyotype	Terminal deletion	43745129-51224208	7,479,080	MLPA	*De novo*	None
SH14	Female	aCGH, FISH	Unbalanced translocation	50267252-51224208	956,957	MLPA	*De novo*	None
SH15	Female	SNP microarray	Terminal deletion	45902119-51224208	5,322,090	MLPA	*De novo*	None
SH16	Female	CMA, FISH	Terminal deletion	42918711-51224208	8,305,498	MLPA	Father negative	None
SH17	Female	SNP microarray	Terminal deletion	45583935-51224208	5,640,274	MLPA	*De novo*	None
SH18	Female	aCGH, FISH	Terminal deletion	50077362-51224208	1,146,847	MLPA	*De novo*	None
SH19	Male	FISH	Interstitial deletion	48551989-51206201	2,654,212	MLPA	Father negative	None
SH20	Male	aCGH	Terminal deletion	51083118-51224208	141,091	MLPA	*De novo*	None
SH21	Male	aCGH	Terminal deletion	45428606-51224208	5,795,603	MLPA	*De novo*	None
SH22	Male	FISH	Terminal deletion	44800014-51224208	6,424,195	MLPA	*De novo*	None
SH23	Male	Karyotype, FISH	Unbalanced translocation	44023173-51224208	7,201,036	MLPA	*De novo*	None
SH24	Male	Karyotype, aCGH	Ring 22	43218614-51224208	8,005,595	MLPA	*De novo*	None
SH25	Male	aCGH, FISH	Terminal deletion	46787434-51224208	4,436,775	MLPA	*De novo*	None
SH26	Male	Karyotype, FISH	Ring 22	49460840-51224208	1,763,369	MLPA	*De novo* deletion; maternal balanced translocation, 46,XX, t(1;6)(p13.3;q22,2)	None
SH27^a^	Male	SNP microarray	Terminal deletion	51115526-51234443	118,917	no DNA, validated in a clinical lab	*De novo* deletion; maternal balanced translocation, 46,XX, t(11;22)(q23;q11.2)	None
SH28	Female	aCGH, FISH	Terminal deletion	45705241-51224208	5,518,968	MLPA	*De novo*	None
SH29	Male	Sequencing	*SHANK3* frameshift mutation (c.2497delG)	No 22q13 deletion detected		Sanger sequencing	*De novo*	None
SH30	Female	FISH	Terminal deletion	49004395-51224208	2,219,814	MLPA	Father negative	None
SH31	Male	aCGH	Terminal deletion	42822943-51224208	8,401,266	MLPA	Mother negative	None
SH32	Male	Sequencing	*SHANK3* nonsense mutation (c.1527G>A)	No 22q13 deletion detected		Sanger sequencing	*De novo*	*De novo* 17q12 microduplication (chr17:34815184–36249059)^b^

^a^No blood was available for participant SH27 or his parents so we present the findings of the clinical report, which used the Affymetrix 6.0 platform. ^b^Reported previously [45]. aCGH, array comparative genomic hybridization; CMA, chromosomal microarray analysis; FISH, fluorescent *in situ* hybridization; hg, human genome version; MLPA, multiplex ligation-dependent probe amplification; SNP, single nucleotide polymorphism.

### Autism spectrum disorder evaluation

We used standardized methods including the ADI-R, ADOS-G and DSM-IV diagnosis based on psychiatric evaluation to evaluate for ASD symptoms in the sample (Table 2). Classifications of ‘autism’, ‘autism spectrum’ and ‘not ASD’ were used in evaluating the diagnostic data. Patients classified as ‘autism’ met DSM-IV criteria for autistic disorder on psychiatric intake, met autism spectrum or autism cutoffs on ADOS-G, Module 1 revised algorithm, and met cutoffs for autism on at least two of three domains on the ADI-R. Patients classified as ‘autism spectrum’ met DSM-IV criteria for pervasive developmental disorder not otherwise specified, autism spectrum cutoffs on the ADOS-G, and met cutoffs on at least two of three domains of the ADI-R. Twenty-four patients (75%) met full criteria for an autism classification based on these criteria. An additional three patients (9.3%) met criteria for autism spectrum. Five patients (15.6%) did not meet study criteria for an ASD (Additional file 1: Table S1). In approximately 50% of cases, the ADOS-G, ADI-R and DSM-IV were in full agreement. The ADOS-G and DSM-IV diagnosis, which considered all sources of information, were in agreement in all but one case where the participant (SH2) met DSM-IV criteria for autistic disorder but only autism spectrum criteria on the ADOS-G. By contrast, the ADI-R yielded ‘autism’ classifications in four out of five ‘not ASD’ patients (that is, patients who did not meet DSM-IV or ADOS-G criteria for an ASD). In nine ‘autism’ cases, the ADI-R was below threshold on one out of three domains, typically the repetitive behavior/restricted interests domain (Domain C).

**Table 2:** Autism spectrum disorder and intelligence quotient diagnostic classifications

	**N**	**%**
**Consensus ASD diagnosis (n = 32)**		
Autism	24	75
Autism spectrum	3	9.4
Not ASD	5	15.6
**Nonverbal IQ classification (n = 30)**		
Average (IQ 100–110)	1	3.3
Mild intellectual disability (IQ 50–55 to 70)	3	10
Moderate intellectual disability (IQ 35–40 to 50–55)	3	10
Severe intellectual disability (IQ 20–25 to 35–40)	7	23.3
Profound intellectual disability (IQ <20-25)	16	53.3

ASD, autism spectrum disorder; IQ, intelligence quotient.

Results from the ADOS-G indicated that 23 participants met criteria for autism and four for autism spectrum (Additional file 1: Table S1). Five participants (age range 2.9 to 25.9 years old) were below thresholds for an autism spectrum classification on the ADOS-G. We also calculated calibrated severity scores [46] in the current sample to provide an estimate of ASD severity compared to reports of severity scores in children with ASD and ID in prior research. The mean calibrated severity score for patients classified as ASD or autism in our sample was 7.07 (SD 1.82). Severity scores between 4 and 6 represent ‘autism spectrum’ classification, and between 6 and 10 correspond to ‘autism’ classifications. Minimally verbal, school-aged children in a previous study presented with scores in the more severe end of this range [46].

**Figure 2:**
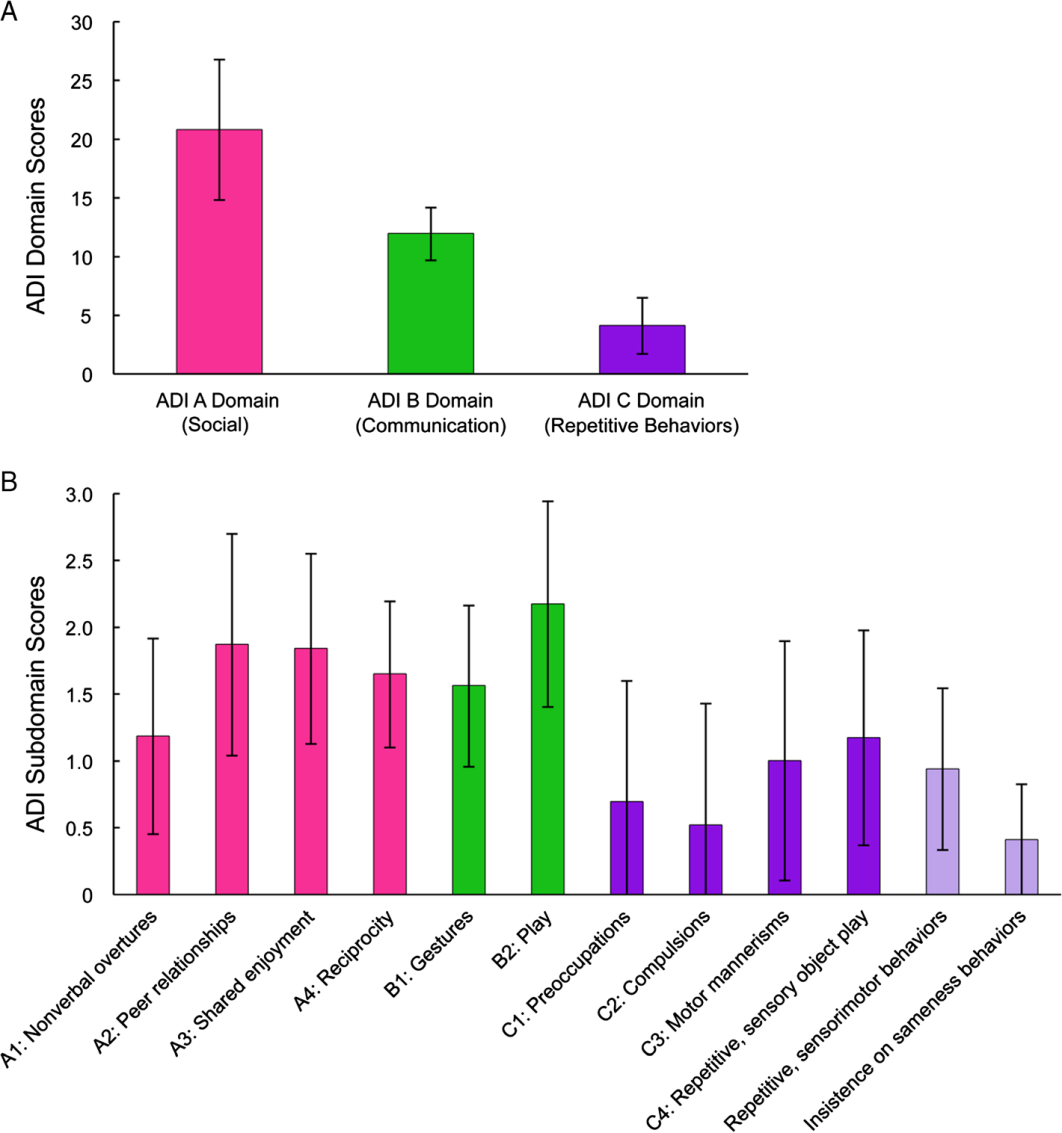
**Mean scores on Autism Diagnostic Interview - Revised. (A)** ADI-R domains and **(B)** subdomains and repetitive behaviors factors (n = 29). Data represent mean ± standard deviation. Cutoff scores for domains are as follows: Social = 10, Communication (nonverbal) = 7, Repetitive behaviors and restricted interests = 3. ADI, Autism Diagnostic Interview.

The ADI-R sample included 30 out of 32 participants. Two of the youngest participants (<25 months) were not administered the ADI-R as it has been shown to have strongest validity in individuals with chronological and mental ages over 18 months. Strict interpretation of the ADI-R indicates that 18 out of 30 participants (60%) met full criteria on the ADI-R. An additional 11 participants met criteria in two of three domains. Eight of these 11 participants had subthreshold scores on the repetitive behaviors domain (that is, 1 to 2 points below). Figure 2 presents the average scores of each domain and subdomain of the ADI-R. The data reflect deficits across ASD symptom domains, including several social communication skills such social reciprocity, engagement, and play skills.

A separate analysis of the ADI-R repetitive behavior domain was conducted to allow comparison with previous research suggesting low rates of repetitive behaviors in 22q13 deletion syndrome [27]. Figure 2 includes data from an analysis of two repetitive behavior factors identified in research on the ADI-R: insistence on sameness, and repetitive sensory and motor behaviors [47]. Interpretation of the ADI-R algorithm alone indicates many children presented with subthreshold levels of repetitive behaviors. However, analysis of all items suggests repetitive and sensory-motor behaviors are reported in all but one participant and represent a range of behaviors seen in idiopathic autism. The item analysis is also consistent with reports from the psychiatric evaluation (see below), which indicated the presence of repetitive behaviors or restricted interests in most participants. The most common repetitive behaviors or restricted interests reported on the ADI-R included unusual sensory interests (n = 21), repetitive use of objects (n = 19), whole body or complex motor mannerisms (n = 15), hand and finger motor mannerisms (n = 13), circumscribed interests (n = 11), unusual sensory sensitivities (n = 11), and negative reactions to changes in personal routines (n = 10). Few reported their children as presenting with unusual preoccupations, having negative reactions to changes in environment, or showing unusual attachment to objects.

### Psychiatric evaluation

A psychiatric review of systems was performed on all participants, and focused primarily on DSM-IV criteria for pervasive developmental disorders. Associated psychiatric and behavioral manifestations were assessed through parent interview, direct child assessment and an autism-focused mental status examination [48] during psychiatric evaluation. On the psychiatric evaluation, the most commonly reported repetitive behaviors included chewing and mouthing objects; hand flapping; jumping; spinning objects; opening and closing doors, drawers and lights; sniffing; breath holding; forced expirations; stereotypic vocalizations; and teeth grinding. Sixteen participants (50%) exhibited current manifestations of hyperactivity and 14 (44%) had episodes of aggression or self-injury. No participants reported current or past motor or vocal tics. Sleep disturbance was also reported in 13 participants (41%).

Behavioral and emotional problems reported in the psychiatric evaluation were also supported by parent ratings on the Vineland Adaptive Behavior Scales. Vineland reports indicated high rates of internalizing behaviors (18.47 ± 2.10), and externalizing behaviors (16.3 ± 2.03). However, item analysis suggests the Vineland does not represent an accurate evaluation of associated symptoms for individuals with *SHANK3* deficiency. The most commonly endorsed ‘internalizing symptoms’ on the Vineland included eating difficulties, sleeping difficulties and poor eye contact, symptoms more likely representing developmental disability rather than psychiatric comorbidity. Items more likely to represent internalizing symptoms consistent with psychiatric illness, such as sadness (n = 3), lethargy (n = 1) and anxiety (n = 4), were endorsed at a low rate in our sample.

We also used information from the psychiatric evaluation and ADI-R to evaluate the presence of skill regression, a developmental pattern commonly reported by parents. Regression was reported to occur as early as 15 months and up to 17 years old. Parents of nine participants (28%) reported skill regression during the psychiatric evaluation, most commonly in the language domain. In at least five of these patients, regression was reported to be associated with onset of seizures. The patient who experienced regression at 17 years of age (SH25) did so after being placed in a residential program, and the regression included loss of language and toileting skills. The ADI-R requires presence of functional, daily spontaneous words prior to loss to capture language regression. Using this more stringent criterion, no participants met criteria for language loss on the ADI-R because of their limited verbal abilities. However, 7 out of 30 (23%) reported loss of skills in other domains, including hand movements (n = 5), other motor skills (n = 3), self help skills (n = 3), play skills (n = 2) and social engagement (n = 4).

#### Cognitive and language testing

**Figure 3:**
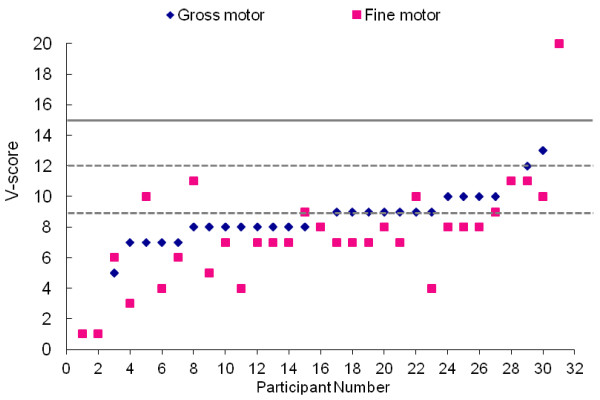
**Motor functioning based on Vineland Adaptive Behavior Scales (n = 32) represented by V scores.** Population mean (15) and standard deviation (3) are represented by solid and dashed lines, respectively.

Results of cognitive testing (Table 2) indicate that the majority of the sample had ID, with only one child scoring in the average range for the nonverbal IQ estimate. Most patients (77%) scored in the severe to profound range of ID. Language was evaluated by clinical intake evaluation, Vineland subscales and, in a subset of the sample, the language domains of the Mullen (Table 3). Five patients (19%) used some words spontaneously to communicate on a regular basis, but none were using spontaneous phrase speech consistently. Standardized testing of language abilities based on Mullen and Vineland scores suggest that the majority of the patients were functioning 2 to 3 SDs below the mean. Figure 3 depicts a scatterplot of receptive and expressive language V scores from the Vineland. Two patients scored in the average range for receptive language abilities, and one patient scored in the average range for expressive language abilities. The latter patient is one of the youngest in the sample (under 2 years old) and as such his score is interpreted with care, given the instability of standardized test scores in very young children with developmental disabilities.

**Table 3:** Language and motor age equivalence scores

	**N**	**Age equivalence months (mean ± SD)**
**Language measures**		
*Receptive language*		
Mullen receptive language	27	11.63±6.25
Vineland receptive language	28	12.40±8.07
*Expressive language*		
Mullen expressive language	27	7.52±4.72
Vineland expressive language	29	9.95±5.98
**Motor ability measures**		
*Fine motor skills*		
Mullen fine motor skills	27	14.89±6.07
Vineland fine motor skills	29	19.62±9.48
*Gross motor skills*		
Mullen gross motor skills	19	17.95±8.52
Vineland gross motor skills	29	21.03±9.02

### Neurological examination

A detailed neurological examination was performed on 16 participants ranging in age from 20 months to 45 years old. The major neurological findings were hypotonia, gait disturbance, and motor planning and coordination abnormalities. Signs often began in early infancy, most commonly manifesting as feeding abnormalities, reported in 7 out of 16 patients (44%). Hypotonia involving limbs and trunk was present in 16 out of 16 patients (100%). At the time of the examination, hypotonia was rated as mild in seven patients and moderate in nine. Signs of upper motor neuron dysfunction, including increased ankle tone, clonus, toe-walking, extensor (Babinski) signs, abnormal posturing of upper extremities and ‘cortical thumbs’, were present in 9 out of 16 patients (56%) evaluated.

**Figure 4:**
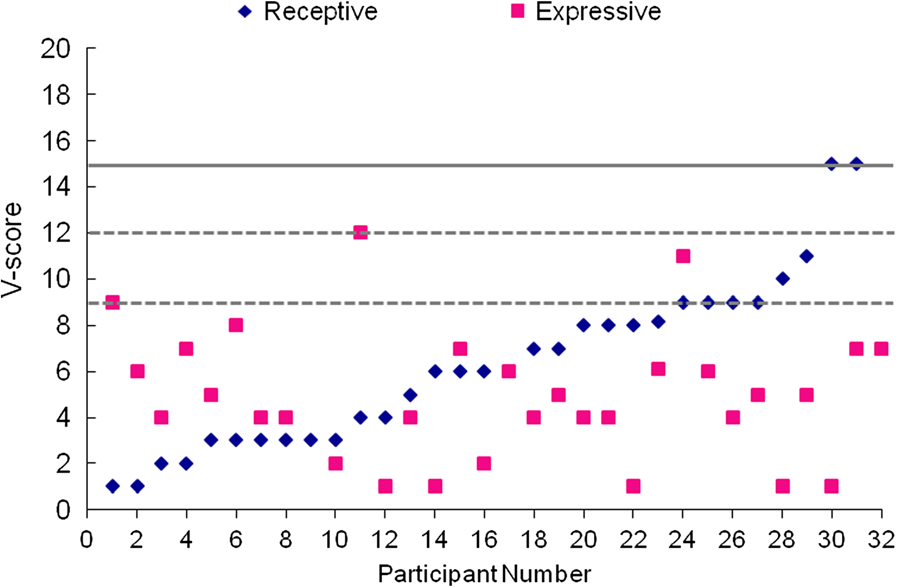
**Language functioning based on Vineland Adaptive Behavior Scales (n = 32) represented by V scores.** Population mean (15) and standard deviation (3) are represented by solid and dashed lines, respectively.

Gait was abnormal in 15 out of 16 patients (94%). Four out of 16 patients were not able to walk independently; three were young children with developmental delay who had not yet walked independently and the fourth patient was an adult who was unable to ambulate after a pelvic fracture and spinal surgery. Gait abnormalities included wide-based stance (n = 5), in-toeing (n = 5), toe-walking (n = 2), steppage (n = 3), feet dragging (n = 2), circumduction (n = 2), knee flexion (n = 4) and upper extremity posturing (n = 2). Motor coordination was found to be grossly abnormal in 14 out 16 patients (88%). In the remaining two patients, gross motor coordination abnormalities were not present, but precise testing was difficult because of cognitive and behavioral limitations. Results of the neurological examination were supported by standardized assessments of gross and fine motor abilities. Scores indicated that patients performed in the 14 to 19 month range on average, regardless of chronological age, on both direct observation (Mullen) and parent report (Vineland) measures of fine and gross motor abilities (Table 3 and Figure 4). While these tests provide insight into the potential extent of the delay, specific patterns of motor deficits have not clearly emerged.

### Dysmorphology evaluation

Thirty-one patients had comprehensive clinical genetic and dysmorphology examinations at Mount Sinai; for one patient we relied on medical record review of an examination at another clinical genetics program. A total of 32 dysmorphic features were evaluated and all patients had at least one abnormal feature (Table 4, Additional file 2: Table S2). The most common dysmorphic feature was the appearance of large fleshy hands, present in 53% of patients, followed by bulbous nose in 47%, long eyelashes in 44%, and minor ear anomalies in 41%. Other frequent features included hypoplastic or dysplastic nails in 34% and full lips in 31%. Accelerated growth has been reported in previous studies [21,22,33,49]. However, only one patient in our study was above the 95th percentile in height. Six children of the 29 participants below the age of 18 had height above the 85th percentile and four children had short stature with height below the 5th percentile. None of the participants with short stature had ring chromosome 22, although a relationship between the two has been previously suggested [23].

**Table 4:** Dysmorphic features identified in the clinical genetic evaluation (N = 32)

**Dysmorphic features**	**N**	**%**	**Estimated frequency from previous reports **[50]
Large, fleshy hands	17	53	>50%
Bulbous nose	15	47	>50%
Long eyelashes	14	44	>50%
Ear anomalies	13	41	>50%
Hypoplastic/dysplastic nails	11	34	>50%
Full lips	10	31	
Epicanthal folds	10	31	>25%
Macrocephaly^a^	10	31	
Dolichocephaly	8	25	>50%
High arched palate	8	25	>25%
Hyperextensibility	8	25	
Full cheeks	8	25	>50%
Periorbital fullness	8	25	>50%
Pointed chin	7	22	>50%
Abnormal spine curvature	7	22	
Wide nasal bridge	5	16	>50%
Long philtrum	5	16	>25%
Sparse hair/abnormal whorl	5	16	
Malocclusion/widely spaced teeth	6	19	>25%
Micrognathia	4	13	
Hypertelorism	4	13	
Short stature^b^	4	13	
Sacral dimple	4	13	>50%
Syndactyly of toes 2 and 3	3	9	
Malar hypoplasia	3	9	
Fifth finger clinodactyly	3	9	<14%
Microcephaly^c^	2	6	
Deep set eyes	2	6	>50%
Accelerated growth^d^	1	3	
Flat midface	1	3	>50%
Ptosis	1	3	>25%
Low set ears	1	3	

^a^ defined as head circumference >97th percentile; ^b^ defined as height <3rd percentile; ^c^ defined as head circumference <3rd percentile; ^d^ defined as height >95th percentile.

### Medical comorbidities

Comprehensive medical record review was performed with records provided by families. EEG was performed in 21 out of 32 cases. Thirteen of 32 participants (41%) had clinical seizures reported by parents, including seven with febrile seizures only (22%; four of these had EEG recordings interpreted as normal; one was interpreted as abnormal but the report was not available for review; two did not receive an EEG), four participants (13%) had non-febrile seizures and two (6%) had both febrile and non-febrile seizures. Among patients with non-febrile seizures, five out of six (83%) had generalized seizures and one (17%) had partial complex seizures; all patients with non-febrile seizures had evidence of EEG abnormalities. In one patient, the febrile seizures were complex seizures and one patient required temporal lobectomy due to uncontrolled seizure disorder. Four patients (13%) had abnormal EEG recordings without reported clinical seizures.

Among other comorbid medical conditions (Table 5), increased pain tolerance and hypotonia were the most common, occurring in 88% and 75% of the patients, respectively. In addition, 17 patients (53%) had a history of recurring infection of the upper respiratory tract, most commonly associated with ear infections in 14 patients (44%) . Two patients also had recurring staphylococcus skin infections with a history of cellulitis. Other common conditions were gastroesophageal reflux disease (44%), sleep disturbance (41%), constipation and/or diarrhea (38%), renal abnormalities (38%), lymphedema (22%), seasonal allergies (19%) and food allergies (16%). Specific renal abnormalities on medical record review included vesicoureteral reflux (n = 4), hydronephrosis (n = 4), renal agenesis (n = 2), extrarenal pelvis (n = 1), dysplastic kidney (n = 1), and bilateral horseshoe kidneys and pyelectasis (n = 1). Only one patient presented with a cardiac defect, aortic regurgitation. Two patients had strabismus and the following conditions affected a single patient each: hypothyroidism, hypertrichosis, vitiligo, intestinal lymphangiectasia, celiac disease, eczema, and esophageal yeast overgrowth with esophagitis.

**Table 5:** Medical comorbidities identified from clinical interviews and medical record reviews (n = 32)

**Medical comorbidity**	**N**	**%**	**Estimated frequency from previous reports **[50]
Increased pain tolerance	28	88	>50%
Hypotonia	24	75	>75%
Recurring upper respiratory tract infections	17	53	
Gastroesophageal reflux	14	44	>25%
Sleep disturbance	13	41	
Seizures (febrile and/or non-febrile)	13	41	>25%
Constipation and/or diarrhea	12	38	
Renal abnormalities	12	38	>25%
Lymphedema	7	22	>25%
Seasonal allergies	6	19	
Food allergies	5	16	
Asthma	3	9	
Strabismus	2	6	>25%
Cardiac abnormalities	1	3	>25%
Hypothyroidism	1	3	5%
Hypertrichosis	1	3	
Vitiligo	1	3	

### Neuroimaging

Medical record review indicated that 28 out of 32 participants (88%) had MRIs performed and 21 out of 28 (75%) had abnormal findings. Reported abnormalities were consistent with those reported in previous studies [24,27,28,36] and included thinning or hypoplasia of the corpus callosum (n = 5), white matter changes (for example, delayed myelination, generalized white matter atrophy, and nonspecific white matter hyperintensities or gliosis) (n = 9), ventricular dilation (n = 7) and arachnoid cysts (n = 5).

### Genotype-phenotype correlations

First, broad domains were used in the analysis of the association between deletion size and clinical features (Table 6). The domains included the total number of dysmorphic features identified by clinical examination, number of medical comorbidities, nonverbal IQ, Vineland language domain V scores, Vineland motor domain V scores and ADI subdomain scores to capture autism symptomatology. The results of the SROC analysis indicated significant associations between larger deletion size and the following variables: number of dysmorphic features (*r*_*s*_ = 0.474), number of medical comorbidities (*r*_*s*_ = 0.402), ADI Social (*r*_*s*_ = 0.466) and ADI Communication (*r*_*s*_ = 0.386) domain scores. In addition, SROC estimates for gross motor skills approached statistical significance (*r*_*s*_ = −0.402). Descriptive review also suggests a possible relationship between deletion size and diagnostic classification based on consensus diagnosis. Mean deletion sizes for non-ASD, autism spectrum and autism groups were 2.28 ± 3.52 Mb, 3.57 ± 2.31 Mb and 4.35 ± 2.76 Mb, respectively. While the number of patients in the non-ASD and spectrum categories is small, the progressive increase in deletion size associated with autism diagnosis may warrant further research in larger samples.

**Table 6:** Association between deletion size and phenotypic variables

**Phenotypic variable**	**N**	**Deletion size**	**BCa confidence interval**^**a**^	
			**Lower**	**Upper**
Number of dysmorphic features	32	0.474^b^	0.145	0.738
Number of medical comorbidities	32	0.386^b^	0.022	0.640
Nonverbal IQ estimate	29	−0.332	−0.640	0.112
Gross motor skills (Vineland)	31	−0.402^c^	−0.728	0.036
Fine motor skills (Vineland)	31	−0.123	−0.473	0.254
Expressive language skills (Vineland)	32	−0.184	−0.531	0.199
Receptive language skills (Vineland)	32	−0.231	−0.553	0.154
Qualitative abnormalities in reciprocal social interactions (ADI-R)	30	0.466^b^	0.073	0.723
Qualitative abnormalities in communication (ADI-R)	30	0.498^b^	0.091	0.740
Restricted, repetitive, and stereotyped patterns of behavior (ADI-R)	30	−0.229	−0.592	0.214

^a^Booststrap estimates and bias corrected and accelerated confidence intervals for Spearman rank order correlations; ^b^significant at 0.05 level (two-tailed test) as confidence interval does not contain 0; ^c^approached significance at 0.05 level (two-tailed test). ADI-R, Autism Diagnostic Interview-Revised; BCa, bias corrected and accelerated confidence intervals; IQ, intelligence quotient.

The association patterns found for medical comorbidities and dysmorphic features were explored further through analysis of each individual feature with deletion size using Mann–Whitney U tests. Significant differences between groups with and without specified features were found for seizures, renal abnormalities, lymphedema, pointed chin and bulbous nose (MWU = 36.00, x^2^ = 5.513, degrees of freedom = 1, *P* = 0.019); however, differences were not significant when correcting for multiple comparisons. Note that patient SH32 carried both a point mutation in *SHANK3* and a 1.4 Mb duplication at 17q12 harboring the *HNF1B* gene previously reported [45], so analyses were run with and without this patient, with similar findings.

Second, we looked at the relationship between major medical findings seen in at least four patients and deletion size to begin to understand whether certain genes are associated with specific phenotypes outside the core phenotypes (see Additional file 3: Table S3). Seizures and abnormal EEG were present in many patients, including those with the smallest deletion or just a point mutation in *SHANK3*. This was also true for gastroesophageal reflux disease, hypotonia, sleep disturbances, abnormal brain MRI and certain dysmorphic features (for example, dolichocephaly, long eyelashes, dysplastic nails, large fleshy hands and full lips). For this reason, it is most parsimonious to attribute these phenotypes to *SHANK3*. By contrast, lymphedema, asthma, cardiac abnormality and renal abnormalities were only observed in larger deletions, consistent with the potential involvement of additional genes in these manifestations.

### Sex differences

The relatively equivalent male:female ratios in *SHANK3* deficiency allowed for analysis of sex differences on phenotyping variables. No differences were found between males (n = 18) and females (n = 14) on deletion size, IQ, autism severity, autism diagnosis, adaptive behavior, number of dysmorphic features or number of medical features.

## Discussion

The aim of this study was to evaluate a serially ascertained sample of children and adults with *SHANK3* deletion or mutation and provide an overview of diagnostic, medical and psychological presentations to guide future research on this syndrome. The results present a picture of *SHANK3* deficiency as a disorder characterized by ID, ASD, predominance of gross motor impairments including gait disturbance and hypotonia, and severe speech delays. Approximately half of the sample also reported a history of abnormal EEG findings and gastroesophageal reflux disease on medical record review.

Patterns of dysmorphic features were not observed in our sample. While all patients presented with at least one dysmorphic feature, the most common feature, large fleshy hands, was only present in 53%. It is also important to note that hand size was within height expectations for the sample and, as such, this feature may represent appearance rather than actual size differences. Other commonly observed dysmorphic features included bulbous nose, long eyelashes and ear anomalies. Our sample also did not present with a pattern of accelerated growth reported in previous studies [21,22,33,49]. These results are consistent with a recent study suggesting that the majority of children with *SHANK3* deficiency have normal growth [51].

The integration of careful clinical evaluation, caregiver report and direct structured observations used in autism research has been shown to increase the validity of ASD diagnoses [52]. To date, published studies have not utilized standard research methods for assessing the presence of ASD in *SHANK3* deficiency. Using the recommended diagnostic methodology, our study found rates of approximately 84% for all ASDs, with rates of 75% for strictly defined autistic disorder and 9.3% for subthreshold symptoms consistent with ASD on the DSM-IV. Rates of autism in our sample were higher than studies relying only on clinical judgment or parent report, which report rates ranging from 26% [29] to 54% [24]. By contrast, studies utilizing at least one standardized assessment tool report rates comparable to the present study. Using the Childhood Autism Rating Scale [53], an investigator rated instrument, Phelan *et al*. [22] found 94% of their sample met criteria for an ASD and Jeffries and colleagues [25] found a rate of ASD of 85% using the Social Communication Questionnaire [32], a caregiver report screening measure.

Autism-specific diagnostic evaluation tools have been reported to possess limited specificity in individuals with severe ID [35,46]. Our findings suggest discrepancies between ADOS-G and ADI-R results for 13 out of 32 participants. In 4 out of 13 patients, the ADI-R yielded an autism classification when both ADOS-G and clinical diagnosis did not support the presence of ASD. In 8 out of 13 cases, the ADI-R did not yield an autism classification as a result of subthreshold levels of repetitive behaviors. Overall, the ADOS-G and DSM-IV clinical diagnoses likely provide a more representative picture of the social communication and repetitive behavior profiles presenting in children with *SHANK3* deficiency and ASD. As previously mentioned, the ADI-R has less validity in populations with chronological and mental ages below 18 months. In our sample, the mean language age equivalence scores were below 12 months and motor age equivalence scores were below 21 months, suggesting a level of ID which may present diagnostic challenges when using the ADI-R.

Importantly, social communication impairments, and repetitive behaviors or restricted interests were observed in this sample of individuals with *SHANK3* deficiency. The most common social communication impairments included social engagement, reciprocity and play. Our results also indicated a significant association between deletion size and scores on social and communication domains of the ADI-R. This finding suggests that the severity of the behavioral phenotype is associated with larger deletion sizes and runs in contrast to results from Sarasua and colleagues [29], who found that individuals with smaller deletion sizes were more likely to have an ASD. However, the presence of ASD in that study [29] was based on parental report and only 26% of the sample was identified as having ASD.

Previous research had suggested a low rate of repetitive behaviors in individuals with 22q13 deletion syndrome [27]. In the repetitive behavior and restricted interest domain of the ADI-R, over half of our sample displayed intense sensory interests and repetitive toy or object play, which are commonly found in individuals with ID alone. However, approximately one-third of the sample also reported compulsive behaviors such as adhering to rigid routines. Further evaluation of repetitive behaviors and restricted interest domains may be an important direction for future research, particularly given the discrepancy between repetitive behaviors reported on the ADI-R and clinical evaluations, as well as the high rates of sensory-related behaviors observed in this sample of individuals with *SHANK3* deficiency. Increasing sample size and improving specificity of measurements of the repetitive behaviors will be important in reconciling published reports.

The severity of the phenotype in *SHANK3* deficiency is highlighted by the results of the standardized evaluations of cognitive, language and functional skills in this study. No participants used phrase speech on a daily basis, and only five used single words to communicate consistently. Scores from direct testing of language and cognitive skills, as well as parent reports on the Vineland, reflect the severe language disability found in most individuals with *SHANK3* deficiency. One of 32 participants had a nonverbal IQ estimate in the average range, with approximately three-quarters of participants performing in the severe to profound range of ID.

The floor effects observed on standardized testing did not allow for comparison of differences within domains (for example, receptive or expressive language). While standardized tests allow comparison of the phenotype relative to general population norms, available tools are hampered by a restricted range in individuals with ID. As such, traditional methods are likely insufficient for the purposes of treatment development or progress evaluations. Development of evaluation tools designed to capture precursors of language and early cognitive signals, such as auditory discrimination and visual attention, are needed in future investigations of *SHANK3* deficiency.

One significant pattern of results relates to the prevalence of motor impairments found across evaluation methods. Motor disabilities were identified in the earliest clinical profiles of *SHANK3* deficiency and their predominance is supported by findings in our sample. The comprehensive clinical evaluation suggests hypotonia as a dominant, early presenting clinical feature, present in 100% of the 16 children who underwent neurological examinations in our sample and described by parents in 75% of the total 32 patients. Motor impairments due to hypotonia may result in feeding difficulties, which are commonly reported as one of the first signs noticed by families in infancy. The neurological evaluation in this study also found high rates of gait abnormalities. Gross motor impairments were moderately associated with deletion size, albeit slightly below the threshold of significance.

Other common clinical features in our sample included gastroesophageal reflux disease, seizures, sleep disturbance, renal abnormalities, and constipation and/or diarrhea. The presence of recurring ear and upper respiratory tract infections was notable and may reflect poor airway protection and sputum clearance due to low muscle tone in *SHANK3* deficiency. It is also possible that *SHANK3* may play an important role in immune function. Immunocytochemical analyses in rats have verified the presence of Shank3 proteins in thymic tissue and suggest a role of Shank3 in the coordination of immune cell signal transduction [54]. Previous case reports have also suggested a link between immune dysfunction and *SHANK3* deficiency [55,56]. Our findings suggest an increased prevalence of recurring infections in our sample of patients with *SHANK3* deficiency and its relationship to *SHANK3* and neighboring genes is an area that should be more carefully explored in the future.

Twenty-eight of our patients had MRIs performed and, among them, 75% had abnormal findings based on clinical reports. The prevalence of structural brain abnormalities in our sample highlights the paucity of data on the abnormal neural systems underlying this syndrome. Understanding how brain structure is affected in *SHANK3* deficiency will be important for future studies. Given that little is known about the neurobiology associated with *SHANK3* deficiency, identification of specific brain abnormalities will aid in more thorough characterization and may provide a critical link between *SHANK3* deficiency and associated behavior.

Significant regression in motor and social skills has also been reported among patients in our sample and in the literature [2,5,30,57,58]. Isolated reports have emerged recently describing significant medical complications of *SHANK3* deficiency in the context of severe cognitive and behavioral regression, including seizure-induced aspiration [2], renal failure [5] and pneumonia [58]. In addition, recent reports of psychiatric comorbidity in individuals with *SHANK3* deficiency suggest that, as patients age, they may be at increased risk for bipolar disorder [30,57,59]. Our results suggest that regression may occur at various ages, across multiple domains, and is often associated with the onset of seizures. Future studies examining the natural history of *SHANK3* deficiency should clarify the nature and extent of regression.

Several studies have examined genotype-phenotype correlations in *SHANK3* deficiency and results are inconsistent [21,23,25,26,28,29,60]. The genotype-phenotype correlations in this study are consistent with at least two previous reports [28,29], indicating a correlation between deletion size and more severe phenotypes. In our sample, larger deletion sizes were associated with a greater number of dysmorphic features and medical comorbidities. In addition, a correlation between larger deletion sizes and social communication impairments associated with autism was found in the present study using standardized research administrations of the ADI-R. Nonverbal IQ, fine motor skills, repetitive behaviors and language abilities did not show an association with size of deletion in the 22q13 region. The associations identified in our analysis are limited by several factors, including sample size. However, should a clear pattern of genotype-phenotype correlations emerge in larger samples, it may delineate a role for other genes and pathways in *SHANK3* deficiency. Replication of potential associations between deletion size and medical comorbidity, social impairments, and possibly motor skills, would also provide valuable information for medical monitoring and treatment planning.

## Conclusions

This study provides additional evidence of the severity of intellectual, motor and speech impairments seen in *SHANK3* deficiency, and highlights the prevalence of ASD symptoms in the syndrome. The findings are presented to guide future investigations seeking to comprehensively map the phenotype of *SHANK3* deficiency. Although our results provide support for the use of a standardized, prospective methodology for evaluating language, psychiatric, cognitive and medical features of *SHANK3* deficiency, they also highlight limitations of available methods and emphasize the need for novel measurement tools in subsequent investigations. Finally, in light of recent reports on adolescence or adult onset psychiatric disturbances [30,57,59] and significant cognitive and behavioral regression [2,5,30,57,58], the need for natural history studies that systematically collect health information is apparent. These studies will facilitate our knowledge of disease progression, facilitate selection of treatment targets, improve medical monitoring, and ultimately improve clinical care across the lifespan of people with *SHANK3* deficiency.

## Abbreviations

ADI-R: Autism Diagnostic Interview – Revised; ADOS-G: Autism Diagnostic Observation Schedule-G; ASD: Autism spectrum disorder; CMA: Chromosomal microarray analysis; CNV: Copy number variant; DSM-IV: Diagnostic and Statistical Manual of Mental Disorders – Fourth Edition; EEG: Electroencephalography; FISH: Fluorescent *in situ* hybridization; Hg: Human genome; ID: Intellectual disability; IQ: Intelligence quotient; Kb: Kilobase; Mb: Megabase; MLPA: Multiplex ligation-dependent probe amplification; MRI: Magnetic resonance imaging; PCR: Polymerase chain reaction; SD: Standard deviation; SNP: Single nucleotide polymorphism; SROC: Spearman rank order correlations.

## Competing interests

JDB has submitted a patent on IGF1 treatment for *SHANK3* deficiency. All other authors declare that they have no competing interests.

## Authors’ contributions

LSo participated in study design, data collection, data analysis and manuscript preparation. AK participated in study design, data collection, data analysis and manuscript preparation. JZ participated in data collection and analysis. TL participated in data analysis. DY participated in data analysis. LSc participated in data collection and analysis. YF performed neurological evaluations. ATW participated in study design, data collection, data analysis and manuscript preparation. CG participated in molecular genetic analysis. EP participated in molecular genetic analysis. DH participated in data collection. DG participated in data collection. BA participated in data collection. JPW performed clinical genetics evaluations. AY performed clinical genetics evaluations. RC participated in study design. WC performed statistical analyses. CB assisted with data interpretation and manuscript preparation. JDB assisted with study design, data interpretation and manuscript preparation. All authors read and approved the final manuscript.

## Supplementary Material

Additional file 1: Table S1 Descriptive and diagnostic data by patient: nonverbal IQ, Vineland Adaptive Behavior Scales, ADI-R, ADOS-G, DSM-IV and consensus diagnosis. Click here for file

Additional file 2: Table S2 Dysmorphic features organized by deletion size. Click here for file

Additional file 3: Table S3 Clinical features and medical comorbidities organized by deletion size. Click here for file
