# Supplementary material for: Prospective investigation of autism and genotype-phenotype correlations in 22q13 deletion syndrome and SHANK3 deficiency
Source: Mol Autism. 2013 Jun 11;4:18. doi: 10.1186/2040-2392-4-18 (PMC3707861; doi:10.1186/2040-2392-4-18)
Supplement: Additional file 1: Table S1 — Descriptive and diagnostic data by patient: nonverbal IQ, Vineland Adaptive Behavior Scales, ADI-R, ADOS-G, DSM-IV and consensus diagnosis. [file 2040-2392-4-18-S1.doc]

**Supplemental Table 1.** Descriptive and diagnostic data by patient: Nonverbal IQ estimate, Vineland Adaptive Behavior Scales, ADI-R, ADOS-G, DSM-IV and consensus diagnosis

| **Subject** | **Age (yrs)** | **NVIQ estimate** | **Vineland Adaptive Behavior Scales-II**  **(Standard Score)** | | | | | **ADI-R**^a^ | | | **ADOS-G Module 1**^b^ | | | | | **DSM-IV** | **Consensus**  **diagnosis** |
| --- | --- | --- | --- | --- | --- | --- | --- | --- | --- | --- | --- | --- | --- | --- | --- | --- | --- |
|  |  | **Standard Score** | **ABC** | **Commu-nication** | **Socializa-tion** | **Daily**  **Living** | **Motor** | **A:**  **Social** | **B:**  **Com** | **C: RRBIs** | **Com Total** | **Social Total** | **Com + Social Total** | **RRBI** | **Severity**  **Score** |  |  |
| SH1 | 7.9 | 6.31 | 57 | 54 | 59 | 54 | 59 | 21 | 13 | 5 | 7 | 12 | 19 | 6 | 10 | Autism | Autism |
| SH2 | 19.2 | 10.43 | 34 | 33 | 45 | 33 | 59 | 24 | 9 | 1 | 3 | 5 | 8 | 1 | 4 | Autism | Autism |
| SH3 | 5 | 19.83 | 53 | 54 | 57 | 51 | 59 | 17 | 13 | 4 | 4 | 11 | 15 | 6 | 8 | Autism | Autism |
| SH4 | 7 | 14.39 | 48 | 45 | 50 | 50 | 51 | 28 | 14 | 2 | 5 | 10 | 15 | 1 | 6 | Autism | Autism |
| SH5 | 1.7 | 25.25 | 79 | 90 | 98 | 75 | 65 | 7 | 10 | 3 | 6 | 12 | 18 | 0 | 6 | Autism | Autism |
| SH6 | 2.8 | 23.46 | 58 | 54 | 66 | 60 | 61 | 19 | 13 | 1 | 7 | 14 | 21 | 1 | 7 | Autism | Autism |
| SH7 | 6.8 | 24.57 | 60 | 52 | 75 | 66 | 59 | 23 | 13 | 1 | 3 | 8 | 11 | 1 | 6 | Autism | Autism |
| SH8 | 3.6 | 57.74 | 65 | 59 | 72 | 66 | 75 | 20 | 8 | 6 | 2 | 3 | 5 | 2 | 1 | not ASD | not ASD |
| SH9 | 4.6 | 10.87 | 45 | 40 | 55 | 43 | 51 | 23 | 14 | 6 | 7 | 14 | 21 | 2 | 9 | Autism | Autism |
| SH10 | 2.9 | 97.42 | 75 | 79 | 83 | 75 | 78 | 5 | 8 | 3 | 2 | 4 | 6 | 1 | 3 | not ASD | not ASD |
| SH11 | 4.1 | 30.86 | 57 | 52 | 63 | 58 | 64 | 19 | 10 | 2 | 4 | 7 | 11 | 4 | 6 | Autism | Autism |
| SH12 | 25.9 | 7.71 | 20 | 21 | 20 | 21 | n/a^c^ | 27 | 14 | 8 | 0 | 4 | 4 | 1 | 2 | not ASD | not ASD |
| SH13 | 14.5 | 3.45 | 27 | 30 | 37 | 25 | 51 | 22 | 13 | 2 | 6 | 11 | 17 | 2 | 7 | Autism | Autism |
| SH14 | 3.9 | 40.17 | 61 | 57 | 75 | 60 | 64 | 11 | 13 | 3 | 3 | 8 | 11 | 0 | 4 | ASD | ASD |
| SH15 | 1.7 | 39.6 | 69 | 71 | 90 | 65 | 65 | 14 | 11 | 0 | 2 | 9 | 11 | 0 | 4 | ASD | ASD |
| SH16 | 7.4 | 15.84 | 52 | 48 | 51 | 57 | 43 | 25 | 14 | 5 | 4 | 12 | 16 | 5 | 7 | Autism | Autism |
| SH17 | 12.2 | 11.6 | 38 | 36 | 38 | 47 | 56 | 22 | 13 | 8 | 7 | 9 | 16 | 2 | 6 | Autism | Autism |
| SH18 | 4.5 | 53.9 | 69 | 59 | 70 | 81 | 78 | 21 | 10 | 3 | 2 | 3 | 5 | 1 | 2 | not ASD | not ASD |
| SH19 | 8.3 | 18.92 | 47 | 40 | 53 | 50 | 59 | 26 | 14 | 5 | 5 | 13 | 18 | 5 | 10 | Autism | Autism |
| SH20 | 10.1 | 23.08 | 51 | 57 | 47 | 50 | 61 | 24 | 14 | 7 | 7 | 7 | 14 | 5 | 9 | Autism | Autism |
| SH21 | 3.8 | 26.67 | 46 | 40 | 57 | 46 | 49 | 25 | 14 | 8 | 4 | 14 | 18 | 1 | 7 | Autism | Autism |
| SH22 | 6.2 | 8.06 | 38 | 36 | 42 | 34 | 43 | 30 | 14 | 6 | 6 | 13 | 19 | 3 | 8 | Autism | Autism |
| SH23 | 11.4 | 11.7 | 32 | 31 | 40 | 35 | 56 | 30 | 14 | 4 | 6 | 14 | 20 | 6 | 10 | Autism | Autism |
| SH24 | 4 | 43.84 | 58 | 52 | 65 | 60 | 64 | 22 | 12 | 1 | 6 | 8 | 14 | 3 | 6 | Autism | Autism |
| SH25 | 45.4 | n/a^d^ | 20 | 21 | 20 | 21 | 22 | 18 | 13 | 2 | 2 | 9 | 11 | 1 | 5 | ASD | ASD |
| SH26 | 8 | 15.72 | 54 | 48 | 57 | 54 | 56 | 24 | 11 | 6 | 4 | 13 | 17 | 4 | 8 | Autism | Autism |
| SH27 | 27.8 | n/a^d^ | 20 | 21 | 20 | 21 | 22 | 18 | 6 | 6 | 6 | 8 | 14 | 4 | 8 | Autism | Autism |
| SH28 | 1.9 | 42.02 | 62 | 62 | 73 | 65 | 61 | n/a^e^ | n/a^e^ | n/a^e^ | 5 | 8 | 13 | 3 | 5 | Autism | Autism |
| SH29 | 7.1 | 16.51 | 49 | 45 | 51 | 52 | 40 | 17 | 10 | 7 | 0 | 2 | 2 | 3 | 2 | not ASD | not ASD |
| SH30 | 3.7 | 15.7 | 57 | 57 | 61 | 66 | 67 | 21 | 11 | 4 | 6 | 12 | 18 | 4 | 8 | Autism | Autism |
| SH31 | 2.1 | 23.53 | 62 | 61 | 76 | 62 | 59 | n/a^e^ | n/a^e^ | n/a^e^ | 5 | 11 | 16 | 5 | 9 | Autism | Autism |
| SH32 | 7.3 | 16.36 | 45 | 42 | 49 | 38 | 59 | 23 | 13 | 6 | 6 | 12 | 18 | 5 | 8 | Autism | Autism |

^a^ ADI-R cutoff scores for autism are: Social = 10, Communication (nonverbal) = 7, Repetitive behaviors and restricted interests = 3

^b^ ADOS-G Module 1 cutoff scores for autism and ASD are, respectively: Communication = 4 and 2, Social = 7 and 4, Total Communication + Social = 12 and 7

^c^ Motor standard scores not calculated because the patient is wheelchair bound

^d^ Nonverbal IQ estimate not calculated because IQ test was not conducted for subject

^e^ ADI-R not conducted because of subject’s age

ABC, Adaptive Behavior Composite (Vineland); ADI-R, Autism Diagnostic Interview-Revised; A: Social, Qualitative Abnormalities in Reciprocal Social Interaction; B: Com, Communication; C: RRBIs, Restricted, Repetitive, or Stereotyped Patterns of Behavior; ADOS, Autism Diagnostic Observation Schedule; Com Total, ADOS-G Module 1 Communication Total; Social Total, ADOS-G Module 1 Reciprocal Social Interaction Total; RRBI, ADOS-G Module 1 Stereotyped Behaviors and Restricted Interests Total; DSM-IV, Diagnostic and Statistical Manual of Mental Disorders-IV; IQ, intellectual quotient; NVIQ, Nonverbal IQ; n/a, not available; yrs, years
