# Supplementary material for: Prospective investigation of autism and genotype-phenotype correlations in 22q13 deletion syndrome and SHANK3 deficiency
Source: Mol Autism. 2013 Jun 11;4:18. doi: 10.1186/2040-2392-4-18 (PMC3707861; doi:10.1186/2040-2392-4-18)
Supplement: Additional file 2: Table S2 — Dysmorphic features organized by deletion size. [file 2040-2392-4-18-S2.doc]

**Supplemental Table 2a.** Dysmorphic features present in more than 25% of participants organized by deletion size

| subject | Deletion size | Large fleshy hands | Bulbous nose | Long eyelashes | Ear anomalies | Hypoplastic/dysplastic nails | Full lips | Epicanthal folds | Macrocephaly | Dolichocephaly | High arched palate | Hyperextensibility | Full cheeks | Periorbital fullness |
| --- | --- | --- | --- | --- | --- | --- | --- | --- | --- | --- | --- | --- | --- | --- |
| 29* | 58572 |  |  |  |  |  |  |  |  | x |  |  |  |  |
| 32* | 58572 |  |  | x | x |  | x | x | x |  |  |  |  |  |
| 10 | 101262 | x |  | x |  | x |  |  |  |  |  |  |  |  |
| 27 | 118917 |  |  |  | x |  | x |  | x |  |  |  |  |  |
| 20 | 141090 | x |  | x |  |  | x | x |  |  |  |  | x |  |
| 14 | 956956 | x |  |  | x |  |  | x |  |  |  | x |  |  |
| 18 | 1146846 |  |  | x |  | x | x |  |  |  |  |  | x |  |
| 8 | 1650084 | x |  |  |  | x |  | x |  |  |  | x |  |  |
| 26 | 1763368 | x | x | x | x | x |  | x |  | x | x |  | x | x |
| 3 | 2109778 |  | x |  | x |  |  |  |  |  |  | x |  |  |
| 9 | 2195476 |  |  |  |  | x |  | x |  |  |  | x |  |  |
| 11 | 2195476 |  |  | x |  |  |  | x |  |  |  |  |  |  |
| 30 | 2219813 |  | x |  |  |  |  |  |  |  |  |  |  | x |
| 1 | 2296660 |  |  |  |  |  |  |  |  |  | x |  |  |  |
| 19 | 2698426 | x | x |  |  | x | x |  |  |  |  |  |  | x |
| 2 | 2779249 |  |  |  | x |  | x |  |  | x | x |  |  |  |
| 7 | 4318675 | x | x |  |  | x |  |  |  |  |  |  | x | x |
| 25 | 4436774 |  |  | x |  |  |  |  |  | x | x |  |  |  |
| 5 | 5080737 | x | x |  | x |  | x |  |  |  |  |  | x | x |
| 15 | 5322089 | x | x | x |  |  |  | x | x |  | x | x |  |  |
| 28 | 5518967 |  |  | x |  |  |  |  | x |  |  |  | x |  |
| 17 | 5640273 | x |  |  |  |  | x |  | x |  |  |  |  |  |
| 21 | 5795602 |  | x |  |  |  |  |  | x | x | x |  |  | x |
| 22 | 6424194 | x | x | x |  |  |  |  |  |  |  |  |  |  |
| 6 | 6796505 | x | x |  |  | x |  | x |  |  |  | x |  |  |
| 4 | 6902567 |  |  | x |  |  |  | x | x |  | x |  |  |  |
| 23 | 7201035 | x |  |  | x | x | x |  |  |  |  |  | x |  |
| 13 | 7479079 | x | x |  | x |  |  |  |  |  |  |  |  |  |
| 24 | 8005594 | x | x |  | x |  | x |  | x |  |  |  |  | x |
| 16 | 8305497 |  | x | x | x | x |  |  | x | x |  | x |  |  |
| 31 | 8401265 | x | x | x | x | x |  |  |  | x | x | x | x | x |
| 12 | 8450476 | x | x | x | x |  |  |  | x | x |  |  |  |  |

* In patients carrying point mutations, the size of the "deletion" corresponds to the size of the *SHANK3* gene.

**Supplemental Table 2b.** Dysmorphic features present in less than 25% of participants organized by deletion size

| Subject | Deletion size | Pointed chin | Abnormal spine curvature | Wide nasal bridge | Long philtrum | Sparse hair/abnormal whorl | Malocclusion/wide spaced teeth | Hypertelorism | Short stature | Sacral dimple | Syndactyly of toes 2-3 | Malar hypoplasia | 5th finger clinodactyly | Accelerated growth | Microcephaly | Deep set eyes | Ptosis | Flat midface |
| --- | --- | --- | --- | --- | --- | --- | --- | --- | --- | --- | --- | --- | --- | --- | --- | --- | --- | --- |
| 29* | 58572 |  |  |  |  |  |  |  | x |  |  |  |  |  |  |  |  |  |
| 32* | 58572 |  |  | x |  |  |  | x |  |  |  |  |  |  |  |  |  |  |
| 10 | 101262 |  |  |  |  | x |  |  |  |  |  |  |  |  |  |  |  |  |
| 27 | 118917 |  | x |  |  |  |  |  | x |  |  |  | x |  |  |  |  |  |
| 20 | 141090 |  |  |  |  |  |  |  |  |  |  |  |  |  |  |  |  |  |
| 14 | 956956 |  |  |  |  |  |  |  |  |  |  |  |  |  |  |  |  |  |
| 18 | 1146846 |  |  |  |  |  |  |  |  |  |  |  |  |  | x |  |  |  |
| 8 | 1650084 |  | x | x |  |  |  |  |  | x | x |  |  |  |  |  |  |  |
| 26 | 1763368 |  |  | x | x | x |  |  |  |  |  |  |  |  |  |  |  |  |
| 3 | 2109778 |  |  |  | x |  |  |  |  |  |  |  | x |  |  |  |  |  |
| 9 | 2195476 |  | x |  |  |  |  |  |  |  |  |  |  |  |  |  |  |  |
| 11 | 2195476 |  |  |  |  |  |  | x |  |  |  |  |  |  | x |  |  |  |
| 30 | 2219813 | x |  |  |  |  |  |  |  |  |  | x |  |  |  |  |  |  |
| 1 | 2296660 |  |  |  |  |  |  |  |  |  | x |  |  | x |  |  |  |  |
| 19 | 2698426 |  |  |  |  |  | x |  |  |  |  |  |  |  |  |  |  |  |
| 2 | 2779249 |  |  |  |  |  | x |  |  |  |  |  |  |  |  |  |  |  |
| 7 | 4318675 |  |  |  |  |  |  |  |  | x |  |  |  |  |  |  |  |  |
| 25 | 4436774 |  | x |  | x |  | x |  |  |  |  |  |  |  |  |  |  |  |
| 5 | 5080737 |  |  | x | x |  |  |  |  |  |  |  |  | x |  |  |  |  |
| 15 | 5322089 | x |  | x |  |  | x |  |  |  |  |  |  |  |  |  |  |  |
| 28 | 5518967 |  |  |  |  |  |  | x |  | x |  |  |  |  |  |  |  |  |
| 17 | 5640273 |  |  |  |  |  |  |  |  |  |  |  |  |  |  |  |  |  |
| 21 | 5795602 | x |  |  |  |  |  |  |  |  | x | x |  |  |  |  | x |  |
| 22 | 6424194 | x |  |  |  |  |  |  |  | x |  |  |  |  |  |  |  |  |
| 6 | 6796505 |  |  |  |  |  |  | x |  |  |  |  |  |  |  |  |  |  |
| 4 | 6902567 | x |  |  |  |  |  |  |  |  |  |  |  |  |  | x |  |  |
| 23 | 7201035 |  |  |  |  | x |  |  | x |  |  |  |  |  |  | x |  |  |
| 13 | 7479079 |  | x |  |  |  | x |  | x |  |  |  |  |  |  |  |  |  |
| 24 | 8005594 |  |  |  | x | x |  |  |  |  |  |  |  |  |  |  | x |  |
| 16 | 8305497 | x |  |  |  |  |  |  |  |  |  |  | x |  |  |  |  |  |
| 31 | 8401265 | x | x |  |  | x |  |  |  |  |  |  |  |  |  |  |  |  |
| 12 | 8450476 |  | x |  |  |  | x |  |  |  |  | x |  |  |  |  |  | x |

* In patients carrying point mutations, the size of the "deletion" corresponds to the size of the *SHANK3* gene
