# Supplementary material for: Prospective investigation of autism and genotype-phenotype correlations in 22q13 deletion syndrome and SHANK3 deficiency
Source: Mol Autism. 2013 Jun 11;4:18. doi: 10.1186/2040-2392-4-18 (PMC3707861; doi:10.1186/2040-2392-4-18)
Supplement: Additional file 3: Table S3 — Clinical features and medical comorbidities organized by deletion size. [file 2040-2392-4-18-S3.doc]

**Supplemental Table 3a.** Clinical features and medical comorbidities present in more than 25% of participants organized by deletion size

| Subject | Deletion size | Repetitive behavior | Pain insensitive | Hypotonia | Hyperactivity | Aggression | GERD | Recurring URTIs | Sleep disturbance | Constipation/diarrhea | Seizures | Febrile seizures | Renal abnormalities |
| --- | --- | --- | --- | --- | --- | --- | --- | --- | --- | --- | --- | --- | --- |
| 29* | 58572 | x | x | x | x | x | x | x | x | x | x |  |  |
| 32* | 58572 | x | x |  | x |  |  |  |  |  |  |  |  |
| 10 | 101262 | x | x |  | x |  |  | x |  |  |  |  |  |
| 27 | 118917 | x | x |  | x | x | x |  | x | x | x |  |  |
| 20 | 141090 | x | x | x | x |  |  | x | x | x |  |  |  |
| 14 | 956956 | x | x | x |  |  | x | x |  |  |  |  |  |
| 18 | 1146846 | x | x |  | x | x |  |  | x |  |  | x |  |
| 8 | 1650084 | x | x | x | x | x | x | x | x |  |  |  |  |
| 26 | 1763368 | x | x | x | x | x | x | x |  |  |  |  | x |
| 3 | 2109778 | x | x | x | x | x | x |  |  |  |  |  | x |
| 9 | 2195476 | x | x | x |  |  | x | x |  | x |  |  |  |
| 11 | 2195476 | x | x | x | x |  |  |  | x | x |  |  |  |
| 30 | 2219813 | x | x | x |  |  |  |  |  |  |  |  |  |
| 1 | 2296660 | x | x | x | x |  | x |  | x | x |  |  | x |
| 19 | 2698426 | x | x | x | x |  |  | x |  |  |  |  |  |
| 2 | 2779249 | x |  | x | x | x |  | x |  |  |  |  |  |
| 7 | 4318675 | x | x | x | x |  |  | x |  |  | x |  |  |
| 25 | 4436774 | x | x |  |  | x |  |  |  |  |  |  |  |
| 5 | 5080737 | x |  | x |  |  |  | x |  |  |  |  |  |
| 15 | 5322089 | x |  | x |  |  |  |  |  | x |  |  |  |
| 28 | 5518967 | x | x | x |  |  | x |  | x |  |  | x | x |
| 17 | 5640273 | x | x | x |  | x |  |  | x |  |  | x | x |
| 21 | 5795602 | x | x | x |  |  |  |  |  |  | x | x | x |
| 22 | 6424194 | x | x | x |  |  |  | x |  | x |  | x | x |
| 6 | 6796505 | x |  | x |  | x | x |  |  | x |  |  | x |
| 4 | 6902567 | x | x | x | x | x | x |  |  | x | x |  | x |
| 23 | 7201035 | x | x |  |  |  | x | x |  | x | x | x |  |
| 13 | 7479079 | x | x | x |  |  | x | x |  |  |  | x | x |
| 24 | 8005594 | x | x |  | x | x |  | x | x |  |  | x | x |
| 16 | 8305497 | x | x | x |  | x | x |  | x |  |  |  | x |
| 31 | 8401265 | x | x | x |  |  |  | x | x | x |  |  |  |
| 12 | 8450476 | x | x |  |  | x |  | x | x |  |  | x |  |

* In patients carrying *SHANK3* point mutations, the size of the "deletion" corresponds to the size of the *SHANK3* gene

GERD, gastroesophageal reflux disease; URTIs, upper respiratory tract infections

**Supplemental Table 3b. Clinical features and medical comorbidities present in less than 25% of participants organized by deletion size**

| Subject | Deletion size | Lymphedema | Seasonal allergies | Food allergies | Asthma | Strabismus | Recurring cellulitis | Hypothyroidism | Vitiligo | Celiac disease | Reactive airway syndrome | Eczema | Esophageal yeast overgrowth | Hypertrichosis | Cardiac abnormality | Lymphagiectasia |
| --- | --- | --- | --- | --- | --- | --- | --- | --- | --- | --- | --- | --- | --- | --- | --- | --- |
| 29* | 58572 |  |  |  |  |  |  |  |  |  |  |  |  |  |  |  |
| 32* | 58572 |  |  |  |  |  |  |  |  |  |  |  |  |  |  |  |
| 10 | 101262 |  | x |  |  |  |  |  |  |  |  |  |  |  |  |  |
| 27 | 118917 |  |  |  |  |  |  |  |  | x |  |  |  |  |  |  |
| 20 | 141090 |  |  |  |  |  |  |  |  |  |  |  |  |  |  |  |
| 14 | 956956 |  |  |  |  |  |  |  |  |  |  |  |  |  |  |  |
| 18 | 1146846 |  |  |  |  |  |  |  |  |  |  |  |  |  |  |  |
| 8 | 1650084 |  |  | x |  |  |  |  |  |  |  |  |  |  |  |  |
| 26 | 1763368 |  | x |  | x |  |  |  |  |  |  | x |  |  |  |  |
| 3 | 2109778 |  |  |  |  |  |  |  |  |  | x |  |  |  |  |  |
| 9 | 2195476 |  |  |  |  |  |  |  |  |  |  |  |  |  |  |  |
| 11 | 2195476 |  | x |  |  | x |  |  |  |  |  |  |  |  |  |  |
| 30 | 2219813 |  |  |  |  |  |  |  |  |  |  |  |  |  |  |  |
| 1 | 2296660 |  |  | x |  |  |  |  |  |  |  |  |  |  |  |  |
| 19 | 2698426 |  |  | x |  |  |  |  |  |  |  |  |  |  |  |  |
| 2 | 2779249 |  | x |  |  |  |  |  |  |  |  |  |  |  |  |  |
| 7 | 4318675 |  |  |  |  |  |  |  |  |  |  |  |  |  |  |  |
| 25 | 4436774 | x |  |  |  |  |  |  |  |  |  |  |  | x |  |  |
| 5 | 5080737 |  | x |  |  |  |  |  |  |  |  |  |  |  |  |  |
| 15 | 5322089 | x |  |  |  |  |  |  |  |  |  |  |  |  |  | x |
| 28 | 5518967 | x |  |  |  |  |  |  |  |  |  |  |  |  |  |  |
| 17 | 5640273 |  |  | x |  | x |  |  |  |  |  |  |  |  |  |  |
| 21 | 5795602 |  |  |  |  |  |  |  |  |  |  |  |  |  |  |  |
| 22 | 6424194 | x |  |  |  |  |  |  |  |  |  |  |  |  |  |  |
| 6 | 6796505 |  |  |  |  |  |  |  |  |  |  |  |  |  |  |  |
| 4 | 6902567 |  |  |  |  |  | x |  |  |  |  |  | x |  |  |  |
| 23 | 7201035 | x | x | x |  |  |  | x |  |  |  |  |  |  |  |  |
| 13 | 7479079 | x |  |  |  |  | x |  |  |  |  |  |  |  |  |  |
| 24 | 8005594 |  |  |  |  |  |  |  |  |  |  |  |  |  |  |  |
| 16 | 8305497 |  |  |  | x |  |  |  |  |  |  |  |  |  |  |  |
| 31 | 8401265 |  |  |  |  |  |  |  |  |  |  |  |  |  | x |  |
| 12 | 8450476 | x |  |  | x |  |  |  | x |  |  |  |  |  |  |  |

* In patients carrying *SHANK3* point mutations, the size of the "deletion" corresponds to the size of the *SHANK3* gene.
